# A rare cause of hematochezia: colonic extranodal marginal zone lymphoma of mucosa-associated lymphoid tissue (MALToma): A case report and literature review

**DOI:** 10.1097/MD.0000000000033869

**Published:** 2023-05-26

**Authors:** Chien-Hung Lu, Wei-Yu Kao, Chun-Chao Chang, Yu-An Kan

**Affiliations:** a Division of Gastroenterology and Hepatology, Department of Internal Medicine, Taipei Medical University Hospital, Taipei, Taiwan; b Department of Internal Medicine, School of Medicine, College of Medicine, Taipei Medical University, Taipei, Taiwan; c TMU Research Center for Digestive Medicine, Taipei Medical University, Taipei, Taiwan; d Taipei Cancer Center, Taipei Medical University, Taipei, Taiwan; e Graduate Institute of Metabolism and Obesity Sciences, Taipei Medical University, Taipei, Taiwan.

**Keywords:** colon, hematochezia, MALToma

## Abstract

Colonic extranodal mucosa-associated lymphoid tissue lymphoma as a cause of hematochezia is rare. Here, we report a case of colonic extranodal marginal zone lymphoma of mucosa-associated lymphoid tissue (MALToma) with presentation of freshy bloody stool and successfully treated by endoscopic mucosal resection.

**Patient concerns:**

This case was a 69-year-old woman with history of hypertension, reflux esophagitis, and peptic ulcer. She had several episodes of hematochezia and thus sought medical attention at the outpatient clinic.

**Diagnoses:**

Colonoscopy revealed a 12-mm semipedunculated lesion in the ascending colon. Histopathological examination and immunochemistry were compatible with colonic extranodal mucosa-associated lymphoid tissue lymphoma.

**Interventions:**

Endoscopic mucosal resection was done for tumor removal and hemoclipping was done to achieve hemostasis.

**Outcomes:**

The patient remained well without recurrence during 3 years of outpatient follow-up.

**Lesson:**

Colonic MALToma is a rare disease, and could present as hematochezia. *En bloc* endoscopic resection could achieve long-term remission. The prognosis of colonic MALToma is excellent with its indolent characteristics.

## 1. Introduction

Extranodal marginal zone lymphoma of mucosa-associated lymphoid tissue (MALToma), first described in 1983 by Isaacson and Wright,^[1]^ is a rare disease, accounting for about 4.3% of non-Hodgkin lymphoma.^[2]^ The most common primary site is the stomach, followed by the eye/adnexa, skin, lung, and salivary glands.^[2,3]^ Colonic MALToma is rarely seen. In recent years, new case studies regarding clinical features, treatment modalities and clinical outcome of colonic MALToma have been proposed. Here, we present a case with presentation of hematochezia and successfully treated with a good outcome. We also conducted a literature view of previous case reports published in PubMed database between 2018 to 2023.

## 2. Case presentation

This 69-year-old woman presented at the outpatient clinic with several episodes of fresh bloody stool. She had a diagnosis of hypertension, reflux esophagitis, and peptic ulcer. However, she had not received follow-up care for 2 years. She reported no use of cigarettes or alcohol. There was no family history of colorectal cancer or familial adenomatous polyposis.

**Figure 1.**
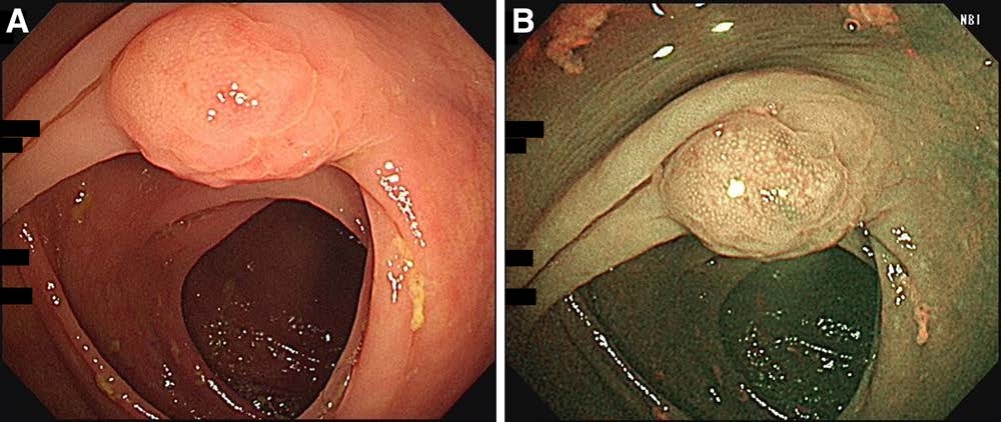
(A) Colonoscopic image of the ascending colon revealed a 12 mm type 0-Isp (semipedunculated) lesion with nodular surface in white light. (B) Colonoscopic image of the lesion revealed a type 1 pit pattern (round pit) in NBI = narrow band image.

**Figure 2.**
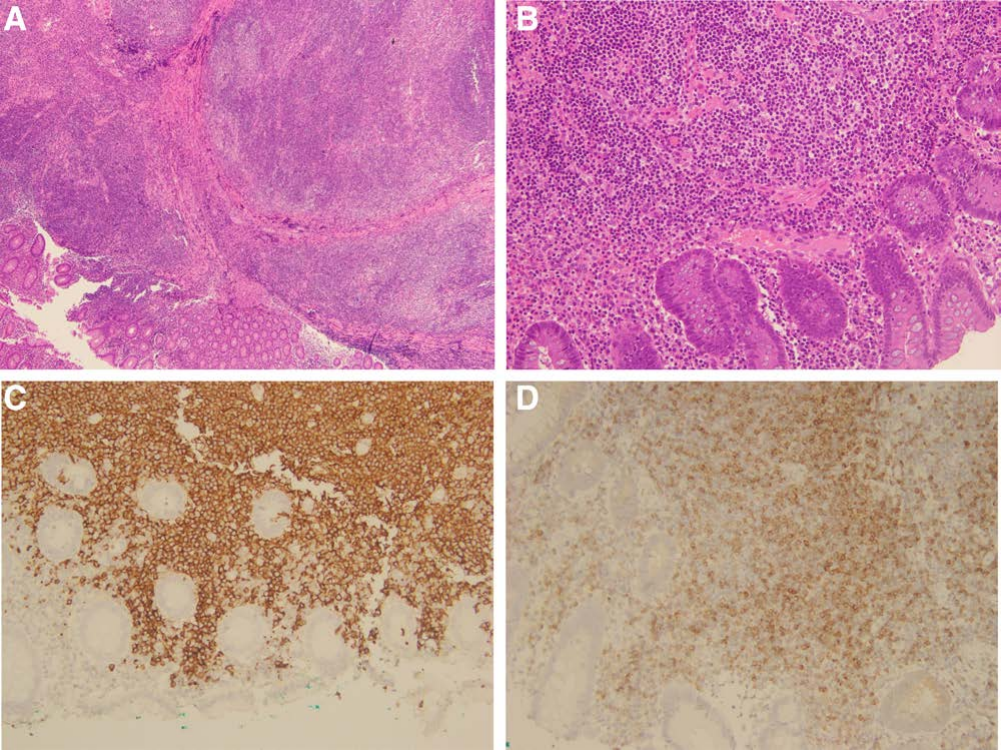
(A and B) The histopathological examination (hematoxylin and eosin staining, 40× and 100×) revealed colon tissue with submucosal infiltration by atypical small to medium-sized lymphoid cells, and pseudofollicular pattern with monocytoid and plasmacytoid cells. (C) By immunohistochemistry, the lymphoid cells were positive for CD20. (D) By immunohistochemistry, the lymphoid cells were positive for BCL-2.

The physical examination was unremarkable. She received a colonoscopic examination performed up to the cecum, which revealed a 12-mm semipedunculated lesion with nodular surface and a type I pit pattern in the ascending colon (Fig. 1A and B). Endoscopic mucosal resection (EMR) followed by hemoclipping was performed to remove the lesion and achieve wound closure. The biopsy specimens revealed colon tissue with submucosal infiltration by atypical small to medium-sized lymphoid cells (Fig. 2A and B). Immunohistochemistry was positive for CD20 (Fig. 2C), BCL2 (Fig. 2D), equivocally positive for CD43, negative for CD3, CD5, cyclin D1, and revealed slightly increased (6%) Ki-67 proliferation index. The pathologic examination was compatible with colonic MALToma. The postprocedural course was uneventful. She had an esophagogastroduodenoscopy simultaneously, which revealed erosive gastritis and suspected intestinal metaplasia. Endoscopic biopsy revealed chronic gastritis with focal intestinal metaplasia and no *Helicobacter pylori* was seen in the superficial gastric pits by hematoxylin and eosin staining. Computed tomography of the chest, abdomen, and pelvis revealed no involvement by lymphoma. A whole-body positron emission tomography was also arranged for tumor screening, revealing negative results. Her hematochezia resolved and repeat colonoscopy confirmed no recurrence of tumor. She remained well during 3 years of outpatient follow-up.

## 3. Discussion

Colonic MALToma is a subtype, accounting for 4.8% of all MALToma.^[2]^ The age-related incidence rate is reportedly 0.57 per 1000,000 person-years.^[2]^ The disease process, similar to gastric MALToma, is typically indolent with usual presentations in localized stages.^[2,4]^ The median age is 68 years old, similar to other sites of MALToma and the disease rate of males is similar to females.^[3]^ The rectum is the most common primary site (74.0%), followed by the right colon (13.6%), and sigmoid colon (8.2%), reported in a review of 50 case reports of colorectal MALToma from 1993 to 2017.^[5]^ The 5-year survival rate is 92.5% in the United States (US) statistics.^[2]^ The tumor recurrence rate is also low (6.8%).^[5]^ The pathogenesis is not well-illustrated, contrary to gastric MALToma in that *H pylori* infection results in chronic inflammation environment, recruitment of B-cells, and *H pylori*-specific tumor-infiltrating T-cells, contributing to proliferation of MALToma.^[6]^ Only 19.2% of colonic MALToma was reported to be *H pylori* positive.^[5]^

The clinical presentations are usually mild or asymptomatic, ranging from incidental finding at screening colonoscopy, abdominal discomfort, a positive fecal occult blood test, bowel habit change, to melena or hematochezia,^[4,5]^ as the presentation of our case. The endoscopic findings are variable. Solitary polypoid lesions were more frequently reported.^[4]^ Flat, elevated, semipedunculated, and ulcerated lesions were also demonstrated.^[4,5]^ A review of case reports showed a median size of 20 mm of tumors, which is appropriate for EMR.^[5]^ Histologically, MALToma comprises of polymorphous lymphoid cells, including monocytoid B-cells, centrocyte-like cells and small lymphocytes. Scattered immunoblasts and centroblast-like cells as well as plasmacytoid differentiation are also seen.^[3]^ Immunohistochemically, MALToma is typically positive for B-cells markers (CD19, CD20, CD79a), BCL2, negative for BCL6, CD5, CD10, and CD23, in accordance with our case. The chromosomal translocations include t(1;14)(p22;q32), t(3;14)(p14;q32), t(11,18)(q21;q21), and t(14,18)(q32;q21), which are involved in the NF-kB pathway.^[6]^

Till now, no consensus guidelines have been established for treatment, which is possibly due to the rarity of the disease. To evaluate the treatment modalities and treatment response in recent published cases, we performed a literature view of previous case reports via a PubMed search of “(Colon OR Colonic OR Colorectal) AND (Mucosa associated lymphoid tissue lymphoma OR Maltoma OR MALT lymphoma)” between January 1, 2018 to February 28, 2023. Cases with concomitant cancer or previous cancer history, congenital disorder, involvement of gastrointestinal tract other than the colon, non-English publications, or incomplete documentation were excluded. A total of 11 cases were included, presented as Table 1.

**Table 1:** Literature view of previous case reports of primary colorectal MALT lymphoma between 2018 to 2023 and the presented case.

Age (yr)	Size (max)	N of Tumor	Stage	Treatment modalities	Status	Follow up period	Ref.
56	0.6 cm	1	I	Endoscopic mucosal resection	Recu-rred	6 mo	^[7]^
77	2.5 cm	3	I	R-THP-COP*1 + BR*3	CR	3 yr	^[8]^
54	0.5 cm	1	I	Endoscopic resection	CR	7 yr	^[9]^
47	1.0 cm	1	I	Endoscopic submucosal dissection	CR	2 yr	^[10]^
50	2.0 cm	1	I	Radiotherapy	CR	2 yr	^[11]^
69	2.5 cm	1	I	Endoscopic polypectomy	CR	1 yr	^[12]^
54	3.0 cm	1	I	EMR followed by ESD	CR	4 yr	^[13]^
60	5.5 cm	1	I	Laparoscopy-assisted ileocecal rese-ction + D2 lymphadenectomy + HP eradication	CR	1 yr	^[14]^
71	5.0 cm	1	I	Endoscopic full-thickness resection	CR	1 yr	^[15]^
80	2.0 cm	1	I	HP eradication	CR	21 mo	^[16]^
72	2.0 cm	1	I	Endoscopic mucosal resection	CR	5 yr	^[17]^
69	1.2 cm	1	I	Endoscopic mucosal resection	CR	3 yr	Present case

BR = rituximab + bendamustine, CR = complete remission, EMR = endoscopic mucosal resection, ESD = endoscopic submucosal dissection, HP = *Helicobacter pylori*, N = number, Ref. = reference, R-THP-COP = rituximab + cyclophosphamide, pirarubicin, vincristine, and prednisone, Stage = Lugano classification.

The treatment modalities include *H pylori* eradication, endoscopic resection, surgery, chemotherapy, radiotherapy, and observation. The treatment response was excellent in the reviewed 11 cases, except for one with recurrence, and rescued by endoscopic submucosal dissection (ESD).^[7]^ Among previous case reports and literature view, some studies showed tumor regression by *H pylori* eradication, even in *H pylori*-negative cases, the mechanism of which is still not elucidated well.^[6,16]^ Eradication of unknown bacteria or antibiotic effects inhibiting tumor development had been proposed.^[5,6]^ The rate of complete remission by *H pylori* eradication (80%) in one review was not inferior to other treatment modalities significantly.^[5]^ In localized stages, EMR, ESD or surgery with *en bloc* resection achieve long-term complete remission in most cases, while radiotherapy is usually used as either a first-line or combined treatment.^[4,11]^ Chemotherapy is more commonly used as a first-line treatment in advanced stages or second-line treatment for recurrence.^[4,8]^ The regimens are various, including rituximab in combination with other agents or rituximab alone. There were also cases having no progression of disease without disease-specific treatment, indicating that a watch-and-wait strategy is possible in selected cases.^[4–6]^ Few cases still experienced disease progression or treatment failure, with the need of second-line therapy.^[4,5]^ Overall, the prognosis of colonic MALToma is excellent with indolent characteristics, usual presentations of localized stages, and good response to treatment modalities.

There were a few limitations in this study. First, only English-written studies were included in the literature view. Publications written in other languages might also provide important information of the disease. Second, cases with MALToma involving multiple sites of gastrointestinal tracts besides the colon were not evaluated. Third, the majority of cases in this study were in localized stages. Future studies including more cases with advanced stages are warranted to obtain a more comprehensive understanding of this disease.

## 4. Conclusion

As illustrated in our case, colonic MALToma has clinical presentation of hematochezia and endoscopic resection either with EMR or ESD with complete removal of tumor results in long-term remission in a localized stage. Our case highlights that physicians’ awareness and alertness to freshy bloody stool or any non-negligible symptom leads to prompt diagnosis of colonic MALToma, and in our case it eventually came with a great outcome after a successful treatment.

## Author contributions

**Supervision:** Wei-Yu Kao, Chun-Chao Chang.

Writing – original draft: Chien-Hung Lu.

Writing – review & editing: Yu-An Kan, Wei-Yu Kao, Chun-Chao Chang.
